# The effect of autogenous tooth bone graft material without organic matter and type I collagen treatment on bone regeneration

**DOI:** 10.1186/s40902-021-00302-w

**Published:** 2021-06-18

**Authors:** Min-Gu Kim, Jung-Han Lee, Gyoo-Cheon Kim, Dae-Seok Hwang, Chul-Hun Kim, Bok-Joo Kim, Jung-Han Kim, Uk-Kyu Kim

**Affiliations:** 1 Department of Oral and Maxillofacial Surgery, School of Dentistry, Pusan National University, Yangsan, 50612 Republic of Korea; 2 Department of Oral Anatomy, School of Dentistry, Pusan National University, Yangsan, 50612 Republic of Korea; 3 Department of Oral and Maxillofacial Surgery, College of Medicine, Dong-A University, Pusan, 49201 Republic of Korea

**Keywords:** Tooth bone graft material, Type I collagen, Autogenous graft

## Abstract

**Objectives:**

The aim of this study is to examine the effect of particulate autogenous tooth graft removed with organic matter and type I collagen addition on bone regeneration and to validate the possibility of useful allograft material for jaw defects.

**Material and methods:**

Autogenous tooth bone maker (Korean Dental Solution® KOREA) made particulate autogenous tooth not including organic matter. We used to the developed tooth grafts for experiment. Cell adhesion test with hemacytometer and energy dispersive X-ray spectroscopy (Supra40 VP®, Carl Zeiss, Germany) analysis about the particulate autogenous tooth and type I collagen were performed. Rabbits were divided into three groups: bone graft with organic matter (OM) removing particulate autogenous tooth group, bone graft with OM removing particulate autogenous tooth and type I collagen group, and a control group. Bone grafting was performed in rabbit’s calvaria. The rabbits were sacrificed at different interval at 1, 2, 4, and 6 weeks after bone grafting for the histopathologic observation and observed the effect of bone regeneration by SEM, H-E & Masson stains, osteocalcin IHC staining.

**Result:**

In vitro cytopathological study showed affinity for cells, cell attachment pattern, and cell proliferation in the order of control group, OM-removed and collagen-treated group, OM-removed particulate autogenous tooth group. The results of the degree of mineralization were opposite to those of the previous cell experimental results, and the OM-removed group, OM-removed group and collagen-treated group were relatively higher than the control group. Histopathologic analysis showed that vascularization and neonatal bone formation were higher in particulate autogenous tooth group with removing OM and with addition of collagen than control group and group of OM removed only. Immunohistochemical analysis showed that osteocalcin (OSC) expression was not observed in the control group, but at 4 weeks groups, OSC expression was observed the OM removed and OM-removed-collagen-treated particulate autogenous tooth, and the degree of expression was somewhat stronger in group of the OM removed and collagen additionally treated particulate autogenous tooth.

**Conclusion:**

Particles that do not contain organic matter, the saint tooth, was responsible for sufficient bone graft material through the role of space maintenance and bone conduction, and further improved bone formation ability through additional collagen treatment. Therefore, research on various extracellular substrates and autologous bone grafting materials is necessary, and through this, it is possible to lay the foundation for a new type of autologous bone grafting material with excellent academic and technical utility.

## Introduction

In various fields of oral-maxillofacial surgery, bone graft procedures for the reconstruction of soft tissue defects are being performed. At the same time, with the growing popularity of dental implant procedures, cases where patients with alveolar bone deficiency are given a combined treatment of implantation and bone defect reconstruction rather than the conventional denture treatment, are increasing.

Bone graft materials are categorized into autogenous, allogenic, heterologous, and alloplastic bone based on the origin and immunological properties. Among them, autogenous bone is the most ideal graft material with respect to stability as it exhibits all three osteogenic, osteoinductive, and osteoconductive potentials, but the limitations are the collectible amount, the patient discomfort and complications after collection [1–3]. To complement these limitations, many studies focused on allogenic or heterologous bone, whose use increased consequently, but these two types also have limitations including the possibility of disease infection and immune rejection, lack of osteogenic potential, and high cost. The disease infection and immune rejection, in particular, are the factors that drive the patients to disfavor the treatment [4–6]. In the case of alloplastic bone, prepared through artificial synthesis of bone-like components, the fact that it has only the osteoconductive function and not the osteogenic or osteoinductive function poses limitations to its clinical application despite partial complementation of the drawbacks of other graft materials and the low cost.

Based on the merits and demerits of the conventional bone graft materials, an ideal graft material should (i) contain a diversity of bone morphogens with osteoinductive property; (ii) not cause immune rejection in patients; and (iii) maintain the stability of the grafted area as well as promoting rapid revascularization and osteogenesis. The ease of collection and affordability should also be taken into consideration.

Recently, autogenous tooth bone graft material (AutoBT) that uses the tooth extracted from the patient, has attained much attention. AutoBT is generally prepared based on the demineralization method using hydrogen chloride (HCl) or nitric acid (HNO_3_), following a process of washing and grinding of the extracted tooth. The strongest advantage of AutoBT is that its histological structure and components resemble those of autogenous bone [7, 8]. As it is an autogenous tissue, AutoBT ensures excellent biocompatibility without immune rejection, which gives emotional stability and satisfaction to the patient. In addition, many recent studies have reported that AutoBT exhibits not only osteoconductive but also osteoinductive potential [9–15].

Among the dental components, enamel comprises 95% inorganic and 0.6% organic matter with 4% water, whereas dentin comprises 70-75% inorganic and 20% organic matter with 10% water. The alveolar bone has a similar chemical composition to enamel and dentin, comprising 65% inorganic and 25% organic matter with 10% water in general. Based solely on the chemical composition, tooth exhibits relatively higher content of inorganic matter and lower content of organic matter than alveolar bone. The inorganic content in tooth and alveolar bone is mostly hydroxyapatite, while the organic matter mostly consists of type I collagen as well as a bone growth factor known as bone morphogenic proteins (BMPs) [7, 8]. Considering the individual roles of each dental component, the type and ratio of the components in a bone graft material is a crucial determinant of the properties of the graft material. Notably, regarding osteoconductive, osteoinductive, and osteogenic potentials, the influence is highly significant. To illustrate, among the components of a bone graft material, when the ratio of organic matter including type I collagen and BMPs falls, the osteoinductive potential is reduced, thereby increasing the time taken for bone formation. Nonetheless, the relative increase in the ratio of inorganic matter with high calcium content leads to enhanced osteoinductive potential with the effect of reduced bone resorption following implantation.

Several studies have found that the conventional AutoBT exhibits an outstanding osteogenic potential [9–15]. However, considering the characteristics of the components of AutoBT, an alteration in the ratio among the components or an addition of an organic matter is anticipated to bring about an enhanced bone repair effect after the bone graft. This study thus applied a new technique to reduce the ratio of organic content in a novel type of AutoBT compared to the conventional one, then grafted the novel AutoBT to the rabbit calvaria to examine the bone repair capability, and during the process, monitored the changes in the bone repair process after the supplementary addition of type I collagen that composes most of the organic content of tooth.

## Materials and methods

### Preparation of AutoBT without organic matter (OM)

The organic substances in the human dental tissue were removed and the tissue was subsequently washed, through the use of autogenous tooth bone maker (Korean Dental Solution®, Busan, Korea) and using sodium chlorate (NaOCl). Next, lipid and protein components in the dental tissue were removed using ethyl alcohol and chloroform, and the tissue was bleached and sterilized using hydrogen peroxide. After 5 times washing, sterilized AutoBT without OM was prepared.

### Cell culture

Human osteosarcoma cell lines, MG63 and HOS, were purchased from ATCC (Rockville, MD, USA). The two different cell lines were immersed in DMEM/F12 1:1 medium and RPMI1640 medium, respectively, then after the addition of 100 μg/ml penicillin/streptomycin, 4 mM l-glutamine, and 10% fetal bovine serum (FBS), the cells were cultured in a 5% CO_2_ incubator at 37 °C.

### Cell adhesion activity measurement

The prepared specimens were sterilized, then after the subculture of 5 × 10^3^ cells, the cells were cultured for 24 h. The specimens were washed twice using phosphate-buffered saline (PBS), after which the attached cells were left in trypsin-EDTA for 5 min at 37 °C. Using the hemacytometer, the cells were counted, and the cell adhesion rate was calculated.

### Cell viability measurement

To measure the cell survival rate, the WST-1 method was followed. A flat specimen on a 6-well plate was prepared, then after the subculture of 5 × 10^3^ cells on the plate, the cells were cultured for 24, 48, and 72 h. Next, 10 μl of WST-1 solution was added to each well for the reaction in an incubator for 2 h, and using the ELISA reader (Tecan, Mannedorf, Swiss), the OD was measured at 450 nm. The analysis was repeated three times for each cell line.

### SEM observation

For the observation of the adhesion pattern of the cells on the specimens, their images were taken using the scanning electron microscopy (SEM; S3500N, HITACHI, Japan). After the subculture of cells on the dental specimens and the addition of medium, the cells were cultured in a 5% CO_2_ incubator at 37 °C for 24 h. Then, the specimens to which cells were attached were fixed using 2.5% glutaraldehyde (Sigma-Aldrich, USA) for 30 min. The specimen preparing was described previously [16]. The specimens were treated with 1% osmium tetroxide (Sigma-Aldrich, USA) for 30 min and dehydrated using ethanol (Sigma-Aldrich, USA) from low concentration to high concentration (70%, 80%, 90%, and 100%). They were then dried using hexamethyldisilazane (HMDS; Sigma-Aldrich, USA). All specimens were immobilized on an aluminum plate and coated using gold sputter in vacuum state. SEM was operated at 15 kV accelerating voltage. All images were photographed at ×2000 and ×5000 magnification, and the resulting images were selected at random for the observation.

### Bone mineralization assay

For the determination of the mineralization ability of osteoblasts on the specimens through quantification of the newly formed layers and crystals on the specimens, the energy dispersive X-ray spectroscopy (EDS, Supra40 VP, Carl Zeiss, Germany) analysis was carried out. The line scan using the EDS and the component analysis for a defined area were carried out at 15 kV, ×40 magnification. The dentin specimens were divided into the top, bottom, left, and right, to which a same size line was specified, then the contents of calcium (Ca) and phosphorous (P) were measured along the line. Also, by defining a same size area around the line at the center, the values of Ca and P were measured within the area. Based on each set of Ca and P values, the Ca/P ratio was obtained and the mineralization ability was assessed.

### Animal experiments

**Fig. 1:**
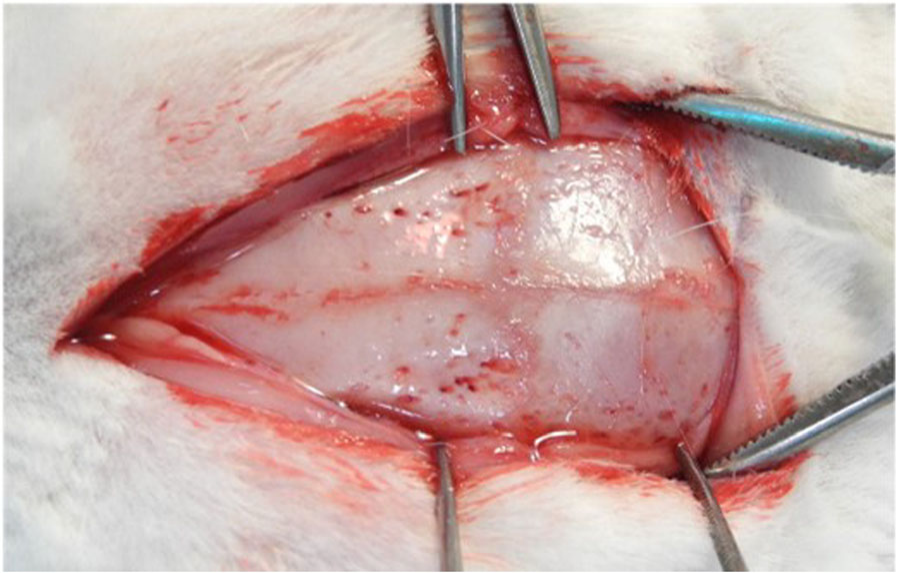
A photo showing the rabbit calvaria after dissection

**Fig. 2:**
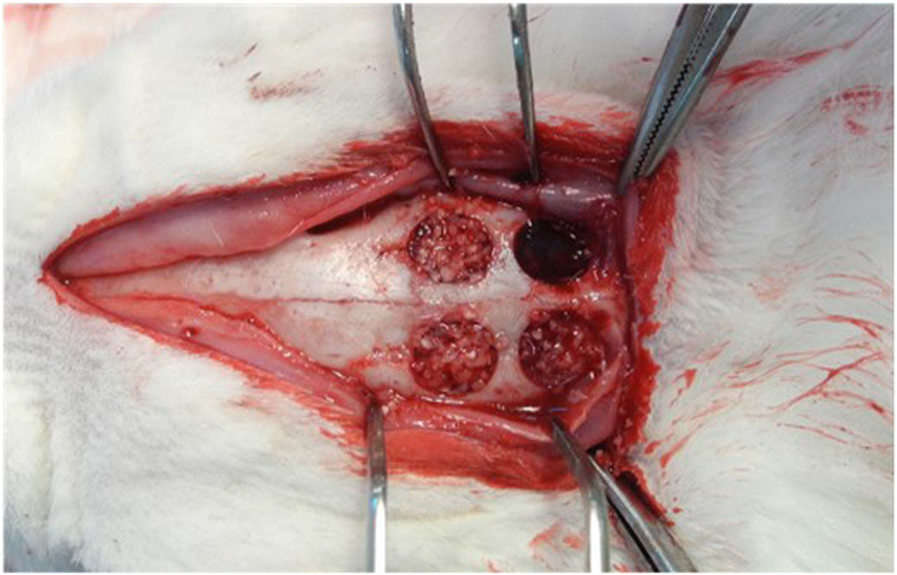
A photo showing four same size defects (8 mm) at the dissected rabbit calvaria after the graft of AutoBT and type I collagen

To the calvaria of 12 rabbits (weight 3.0-3.5 kg, New Zealand), four same size defects (8 mm) were made for the control, experimental group A and experimental group B, and after applying human AutoBT without OM and type I collagen to the defects, three rabbits were sacrificed each at weeks 1, 2, 4, and 6, for monitoring the level of bone formation on the calvarial tissue. No treatment was applied to the defect site of the control; AutoBT without OM was grafted to the defect site of the experimental group A; AutoBT without OM and 100 mg/ml type I collagen were simultaneously grafted to the defect site of the experimental group B. The animals used in the experiments were 8-month-old rabbits between 3.0 kg and 3.5 kg that were bred in a standard laboratory where temperature of 25 ± 1 °C and humidity of 53 ± 5% were maintained, and all experiments and their plans for animal management and diet control were based on the guidelines and regulations of the Institutional Animal Care and User Committee (IACUC; Pusan National University. PNU-2017-1446) (Figs. 1, 2).

#### H-E staining

The bone tissue of each rabbit was fixed in 10% neutral buffered formalin solution for 24 h. The tissue was sliced to 5 μm and hematoxylin and eosin (H&E) staining was performed. The changes in each tissue were monitored using the optical microscope (Pixel link PL-B686 CU, Canada) for the observation of the H-E stained tissue specimens with respect to the level of bone tissue repair at the defect site.

#### Masson’s trichrome staining

The bone tissue of each rabbit was fixed in 10% neutral buffered formalin solution for 24 h. The tissue was sliced to 5 μm and Masson’s trichrome staining was performed. The changes in each tissue were monitored using the optical microscope (Pixel link PL-B686 CU, Canada) for the observation of bone formation in bone tissue repair around the AutoBT graft area at the defect site.

#### Immunohistochemistry

The paraffin section was placed in a 68 °C oven for 1 h, and washed three times with xylene for 7 min each for paraffin removal, then three times with 100% ethanol for 5 min each for xylene removal. The section was then rinsed with 90%, 80%, and 70% ethanol in this order, for 5 min each. The rinsed section was washed with PBS three times for 5 min each to remove alcohol completely. Next, the Mouse and Rabbit Specific HRP/DAB (ABC) Detection IHC Kit (Abcam Inc., Cambridge, UK) was used. Subsequently, the production of osteocalcin was monitored using the Slide Scanner (Axio Scan.Z1, Germany).

### Statistical analysis

Statistical analysis data were expressed ± SD from at least three independent experiments. Statistical analyses used GraphPad Prism version 5.0 for Windows (GraphPad Software, San Diego, CA). A one-way ANOVA was used for Dunnett’s multiple-comparison test in the statistical analysis.

## Results

### Cell adhesion rate

**Fig. 3:**
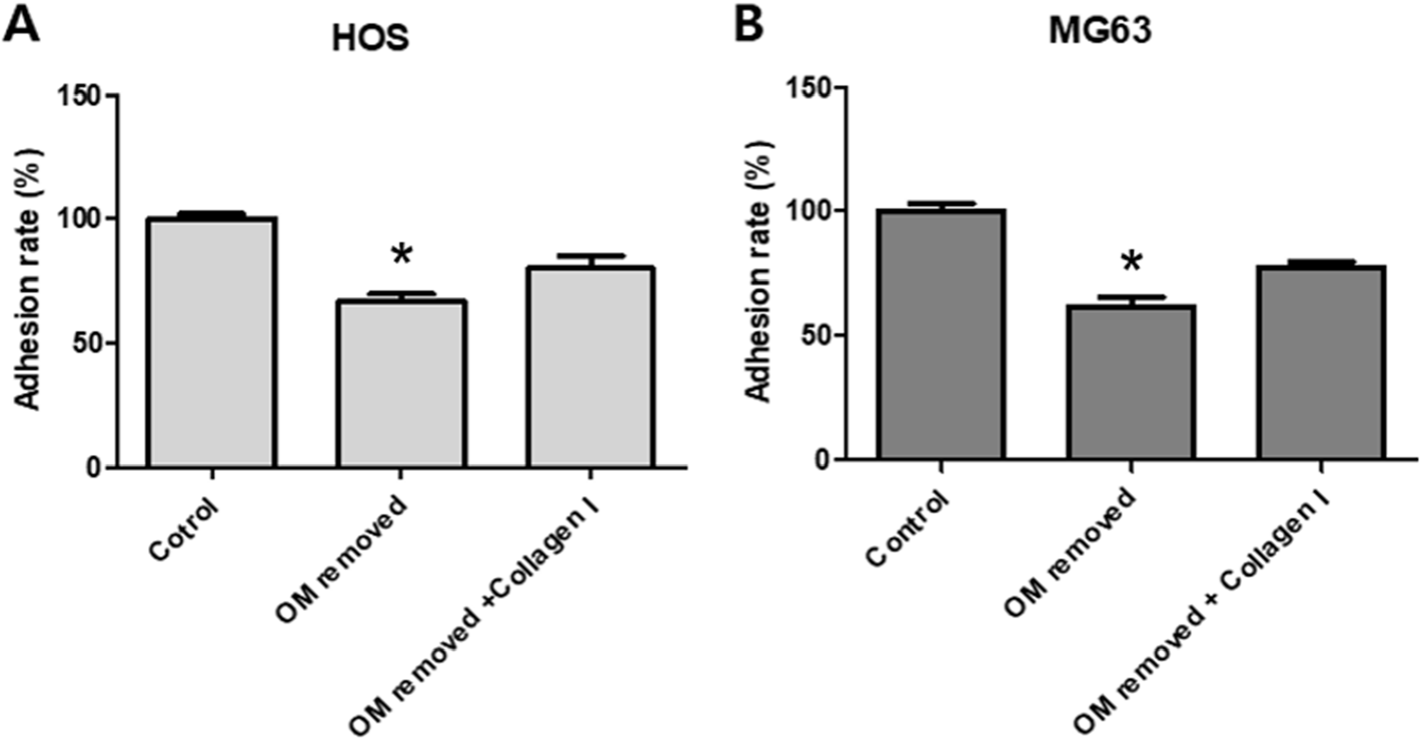
A graph showing the cell adhesion activity of HOS cells (**A**) and MG63 cells (**B**). Each value represents the mean ± S.D. *p < compared with control

First, a cell adhesion test was conducted to examine the affinity for cells of the specimens of human AutoBT without OM prepared using the autogenous tooth bone maker. HOS cell adhesion rate was 100%, 69%, and 81.3%, respectively, in the control with cell adhesion to tooth surface, in the OM-removed group with cell adhesion to AutoBT without OM, and in the OM-removed group to which type I collagen was added. MG63 cell adhesion rate was 100%, 61.4%, and 74.3%, respectively, in the control, in the OM-removed group, and in the OM-removed-collagen-treated group (Fig. 3).

### Cell adhesion morphology

**Fig. 4:**
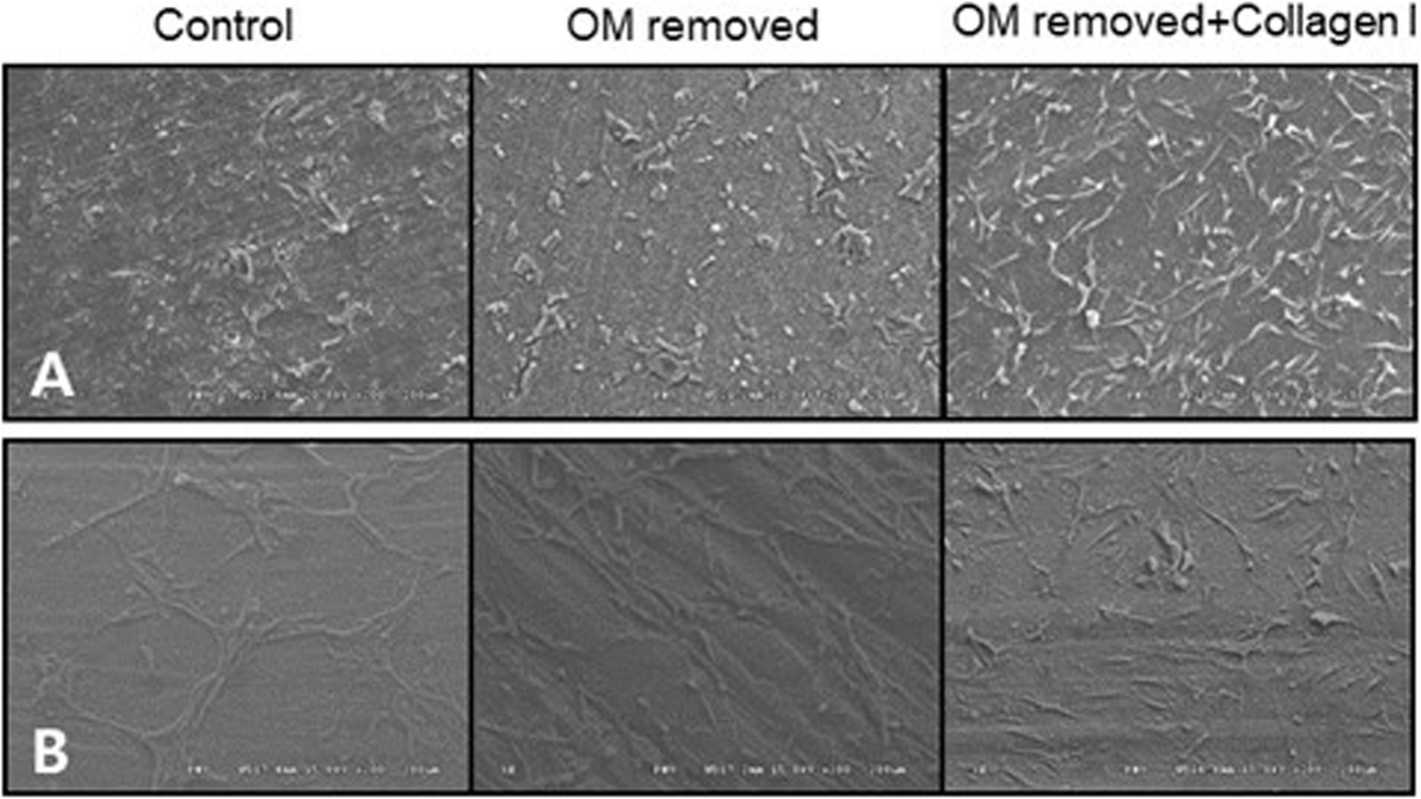
AutoBT-grafted cell adhesion morphology of HOS cells (**A**) and MG63 cells (**B**)

SEM was used for the observation of cell adhesion morphology. MG63 and HOS cells were each divided into three groups: the control, the OM-removed group, and the OM-removed, collagen-treated group, then cultured on tooth specimens. HOS cells showed good surface adhesion in the control specimens, where the cells were seen to maintain a large number of spindle-like protrusions for adhering to the surface, whereas the OM-removed specimens showed weak surface adhesion and only a small number of adhering cells. The OM-removed group to which type I collagen was added showed the cells with spindle-like protrusions but many cells appeared steric in volume due to weak adhesion. MG63 cells, despite different morphology of adhering cells, displayed a similar adhesion pattern to HOS cells (Fig. 4).

### Cell proliferation rate

**Fig. 5:**
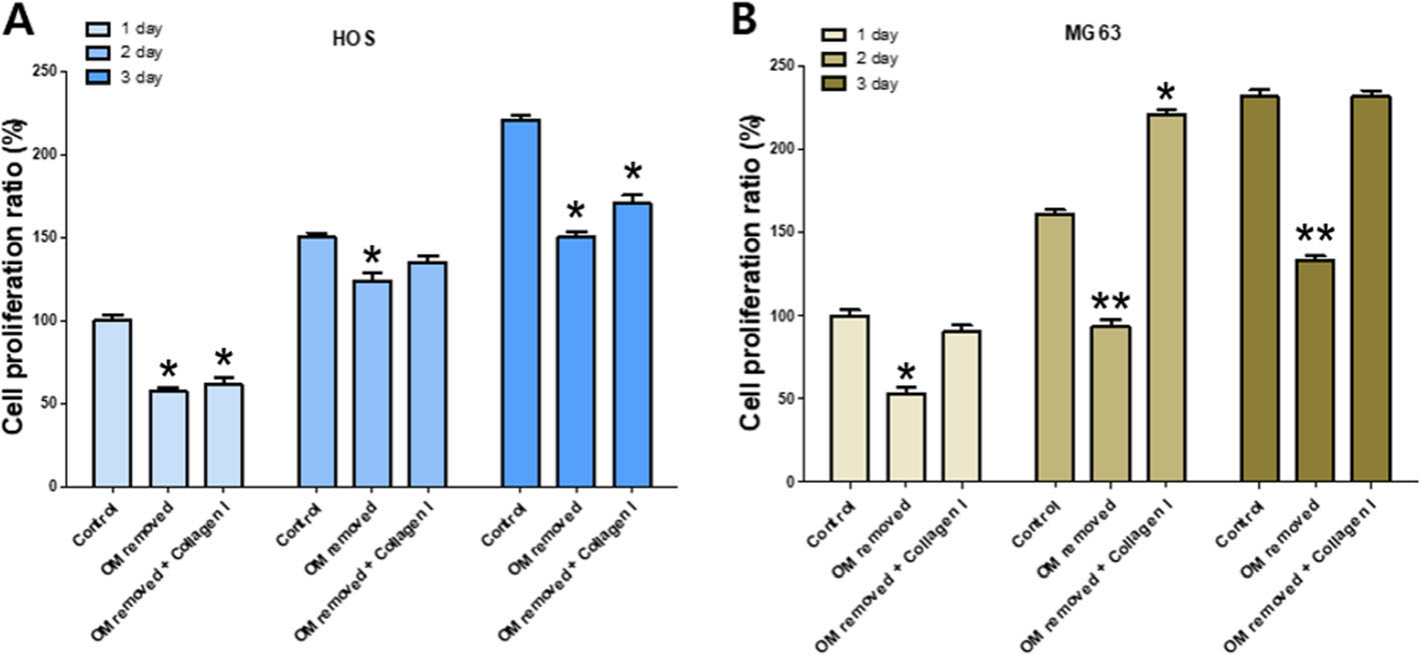
A graph showing the time-dependent cell proliferation rate of HOS cells (**A**) and MG63 cells (**B**). Each value represents the mean ± S.D. *p < 0.05, **p < 0.01 compared with control

The time-dependent cell proliferation rate of HOS cells was 100%, 150%, and 220% in the control; 54%, 124%, and 150% in the OM-removed group; 61%, 135%, and 170% in the OM-removed-collagen-treated group. A significantly higher rate was exhibited by the OM-removed-collagen-treated group in comparison to the OM-removed group (Fig. 5a).

The time-dependent cell proliferation rate of MG63 cells was 100%, 161%, and 232% in the control; 52%, 93%, and 133% in the OM-removed group; 90%, 221%, and 232% in the OM-removed-collagen-treated group. Similar to the HOS cells, the rate was higher in the OM-removed group to which type I collagen was added (Fig. 5b).

### Mineralization assay

**Fig. 6:**
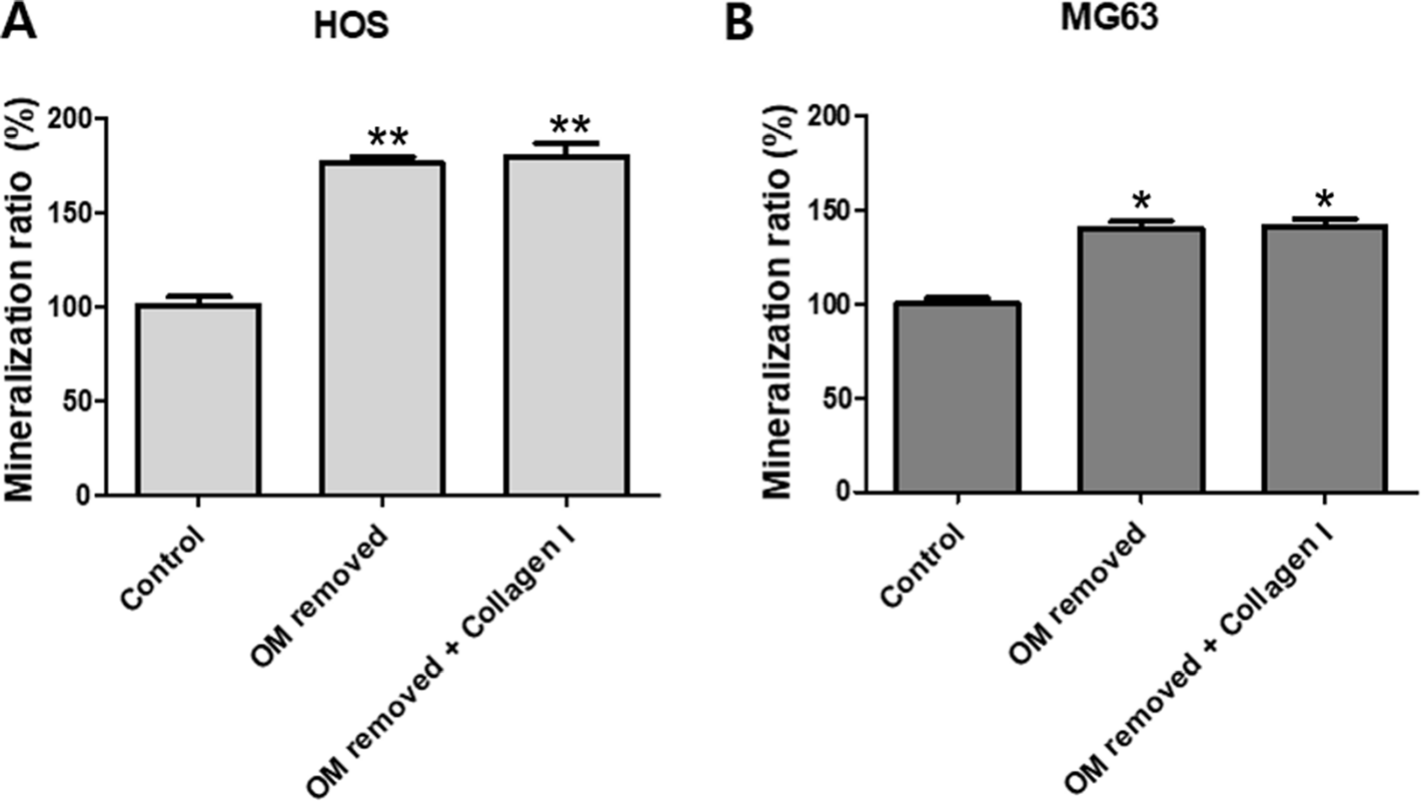
A graph showing the mineralization ratio of HOS cell secretion (**A**) and MG63 cell secretion (**B**). Each value represents the mean ± S.D. *p < 0.05, **p < 0.01 compared with control

The ratio between calcium (Ca) and phosphorous (P) contents in HOS and MG63 cells was estimated, to determine the degree of mineralization in the three groups: control, OM-removed group, and OM-removed-collagen-treated group. HOS cells showed 100% in the control; 176% in the OM-removed group; 180% in the OM-removed-collagen-treated group, while MG63 cells showed 100% in the control; 139% in the OM-removed group; 141% in the OM-removed-collagen-treated group. The degree of mineralization in both cell lines was shown to be higher in the OM-removed and the OM-removed-collagen-treated groups than in the control (Fig. 6).

### Histological findings

#### H-E staining

**Fig. 7:**
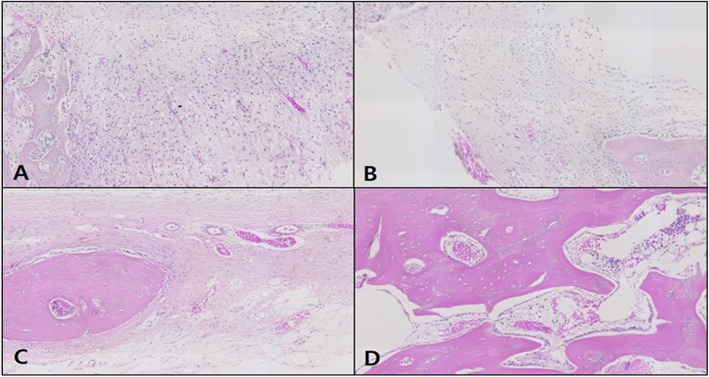
Microphotograph at 1 week (**A**), 2 weeks (**B**), 4 weeks (**C**), and 6 weeks (**D**) after bone graft in control rabbit. H-E. ×50

In the control, the neonatal bone formation was negligible in weeks 1 and 2, while in week 4, a large number of blood vessels and compact connective tissue were observed at the defect site with the start of bone fragment formation upon intramembranous ossification. In week 6, most of the space at the defect site was filled with bone tissue, and compared to the original bone tissue in the vicinity; the neonatal tissue was thin due to insufficient bone matrix. A neat alignment of osteoblasts was seen around the neonatal bone tissue, and an abundance of adipose tissue was prominently observed (Fig. 7).

**Fig. 8:**
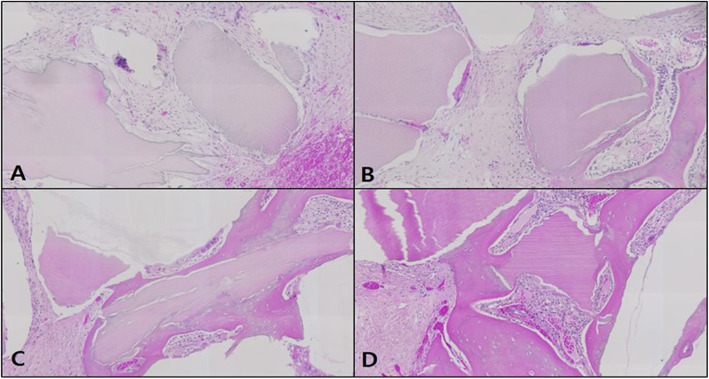
Microphotograph at 1 week (**A**), 2 weeks (**B**), 4 weeks (**C**), and 6 weeks (**D**) after bone graft with organic matter removed particulate autogenous tooth in experimental rabbit. H-E. ×50

In the OM-removed experimental group A, compact connective tissue was observed surrounding the graft material at the defect site in week 1, and a large number of blood vessels were seen to have started forming. In week 2, a large number of osteoclasts were observed around the graft material, with the steady formation of neonatal bone tissue in the area. In week 4, far more concentrated compact connective tissue was observed, compared to week 2, with osteoclasts and osteoblasts around the graft material and neonatal bone formation. In week 6, a lot more blood vessels have formed, and the neonatal bone tissue around the graft material could be seen to have thickened (Fig. 8).

**Fig. 9:**
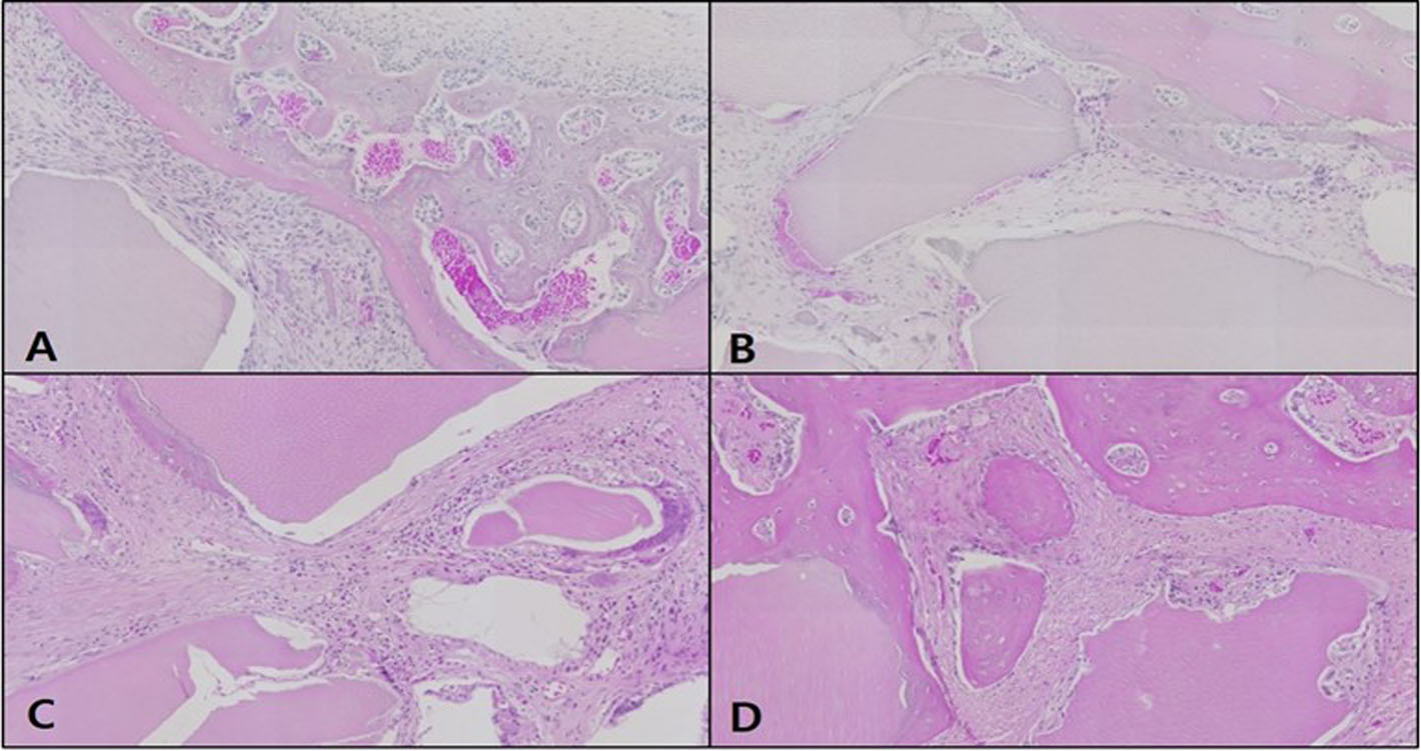
Microphotograph at 1 week (**A**), 2 weeks (**B**), 4 weeks (**C**), and 6 weeks (**D**) after bone graft with organic matter removed particulate autogenous tooth and type 1 collagen in experimental rabbit. H-E. ×50

In the OM-removed-collagen-treated experimental group B, neonatal bone formation and vascularization are predominantly seen under the periosteum at the defect site in week 1. The most substantial difference in week 2 is the high level of vascularization around the graft material in comparison to the other groups, with the neonatal bone tissue under the periosteum having matured further. In week 4, the density of the compact connective tissue around the graft material filling the defect site, could be seen to have increased, while a large number of osteoclasts were observed around the graft material and at the same time, the neonatal bone formation at the end of the graft material. In week 6, many dents were seen to have formed around the graft material, with a high level of neonatal bone formation in the vicinity. A pattern where myriads of osteoblasts were adhering to the area surrounding the graft material could be seen, and the neonatal bone formation was even more elaborate than experimental group A (Fig. 9).

#### Masson’s trichrome staining

**Fig. 10:**
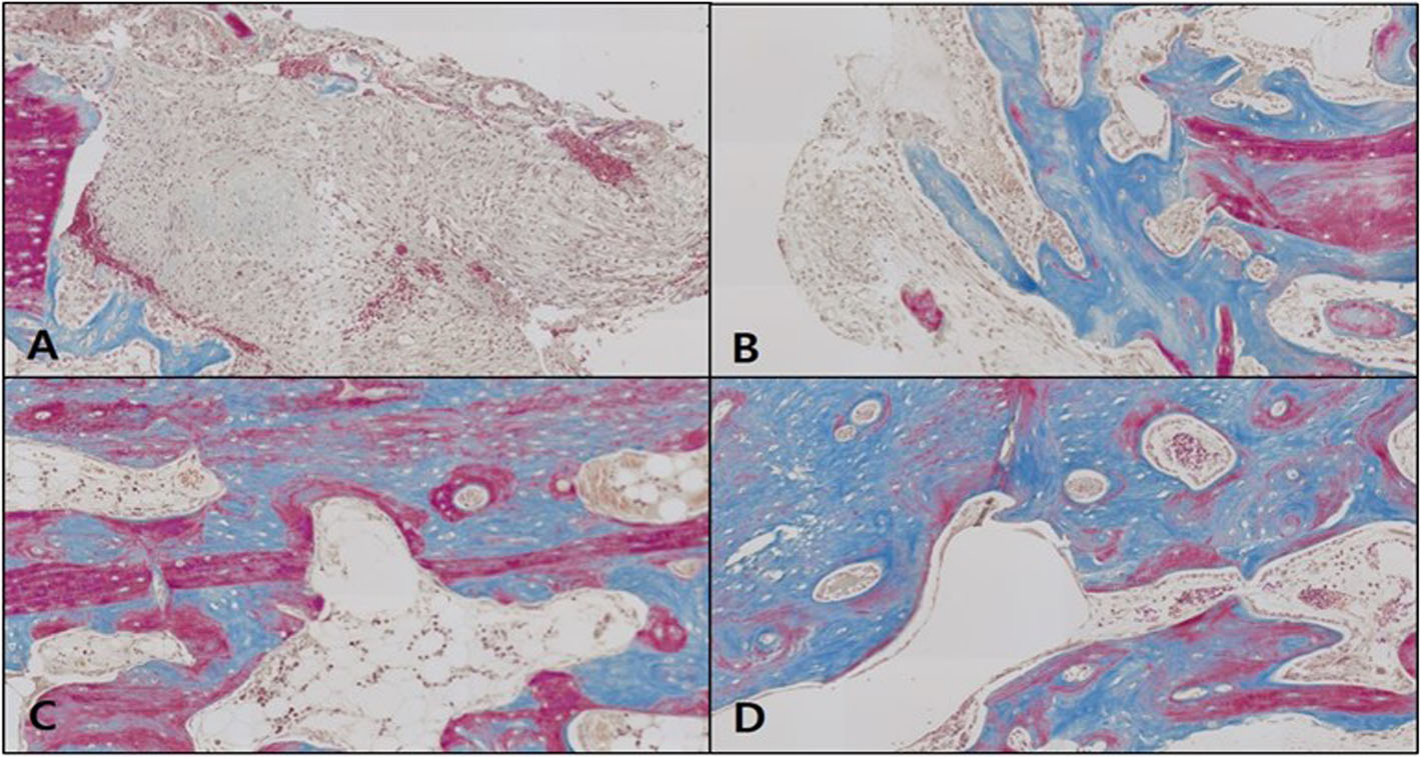
Microphotograph at 1 week (**A**), 2 weeks (**B**), 4 weeks (**C**), and 6 weeks (**D**) after bone graft in control rabbit. Masson. ×50

In the control, the neonatal bone formation was negligible in weeks 1 and 2, while in week 4, the defect site was filled mostly with red, immature bone tissue, and in week 6, the site was filled mostly with blue, mature bone tissue (Fig. 10).

**Fig. 11:**
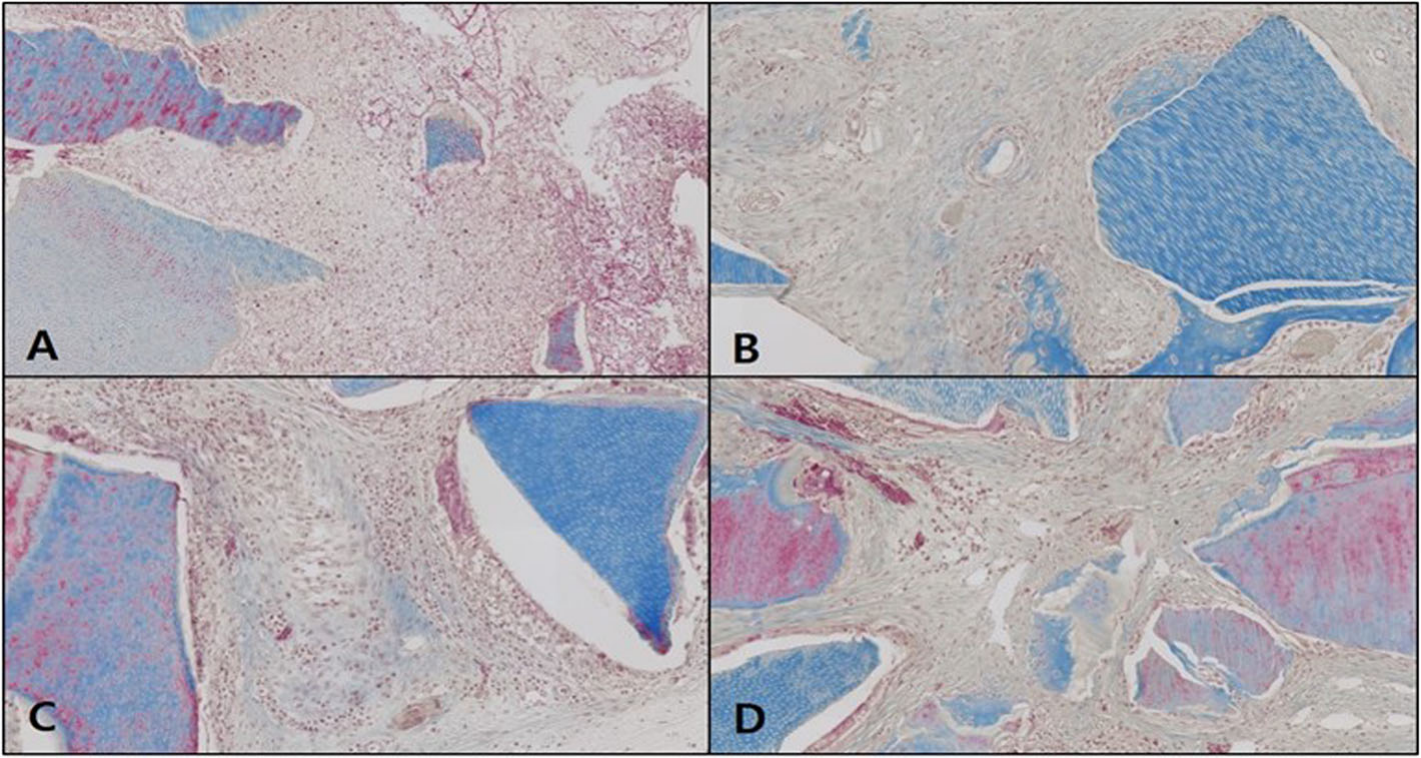
Microphotograph at 1 week (**A**), 2 weeks (**B**), 4 weeks (**C**), and 6 weeks (**D**) after bone graft with organic matter removed particulate autogenous tooth in experimental rabbit. Masson. ×50

In the OM-removed experimental group A, the graft material was seen to be reliably filling the defect site with compact connective tissue immersing the graft material. A similar pattern of neonatal bone formation was seen in weeks 2, 4, and 6. The blue expression of collagen among the compact connective tissue could be observed, but the growth into mature, mineralized bone tissue had not yet occurred (Fig. 11).

**Fig. 12:**
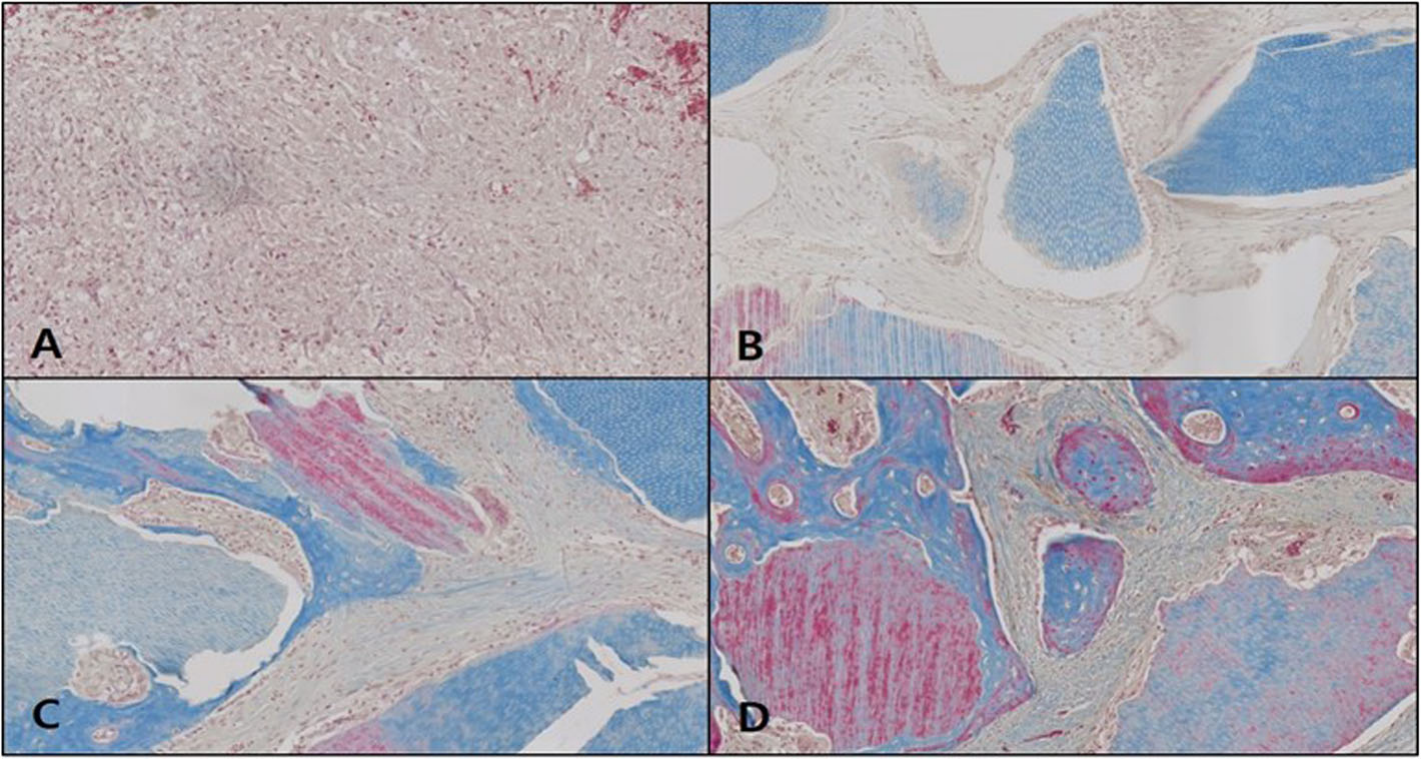
Microphotograph at 1 week (**A**), 2 weeks (**B**), 4 weeks (**C**), and 6 weeks (**D**) after bone graft with organic matter removed particulate autogenous tooth and type 1 collagen in experimental rabbit. Masson. ×50

In the OM-removed-collagen-treated experimental group B, the difference in comparison to other groups was not significant in week 1, whereas in weeks 2 and 4, neonatal bone formation began to be observed in the area surrounding the graft material, and in week 4, collagen expression among the compact connective tissue immersing the graft material could be seen. In week 6, more intensive neonatal bone formation was detected, with stronger expression of collagen among the compact connective tissue (Fig. 12).

#### Osteocalcin immunohistochemical staining

**Fig. 13:**
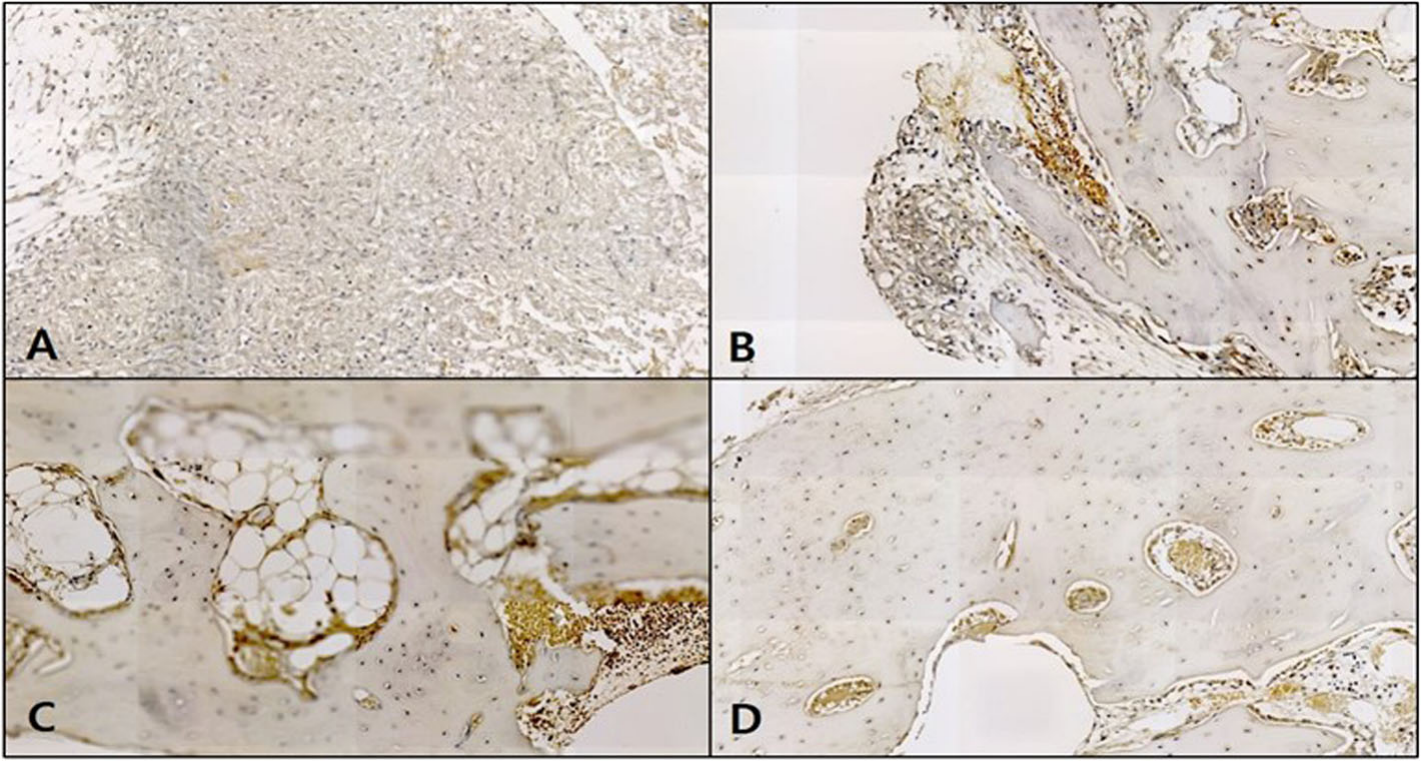
Microphotograph at 1 week (**A**), 2 weeks (**B**), 4 weeks (**C**), and 6 weeks (**D**) after bone graft in control rabbit. Osteocalcin. ×50

In the control, the expression of osteocalcin (OSC) was not observed in weeks 1 and 2 as neonatal bone formation was negligible, and OSC expression was still not observable in weeks 4 and 6. In addition, blood vessels and red blood cells in the form of brown OSC expression were observed (Fig. 13).

**Fig. 14:**
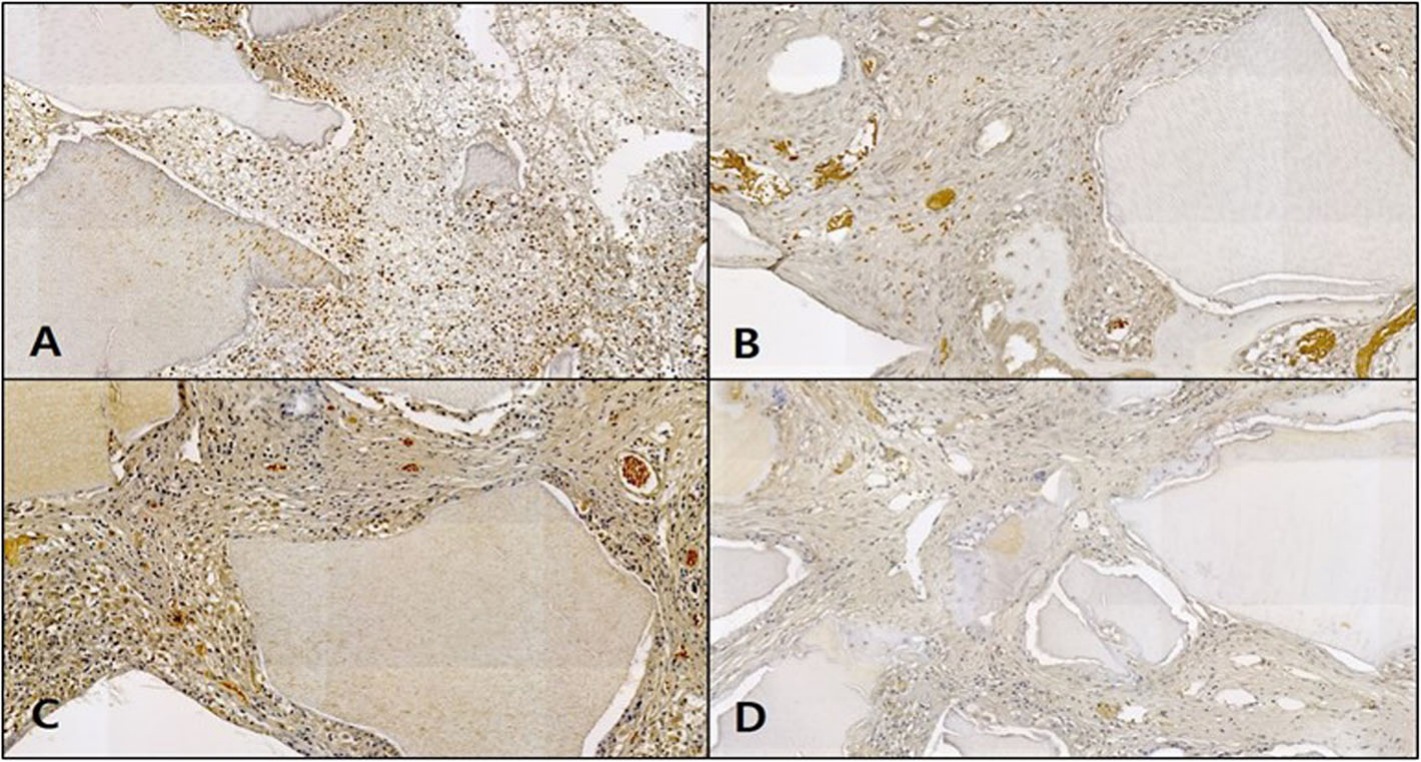
Microphotograph at 1 week (**A**), 2 weeks (**B**), 4 weeks (**C**), and 6 weeks (D) after bone graft with organic matter removed particulate autogenous tooth in experimental rabbit. Osteocalcin. ×50

In the OM-removed experimental group A, OSC expression was absent in weeks 1 and 2, then it appeared in week 4, but disappeared in week 6. OSC expression in neonatal bone tissue was weak overall, and it was observed in parts of blood and the implanted bone graft material (Fig. 14).

**Fig. 15:**
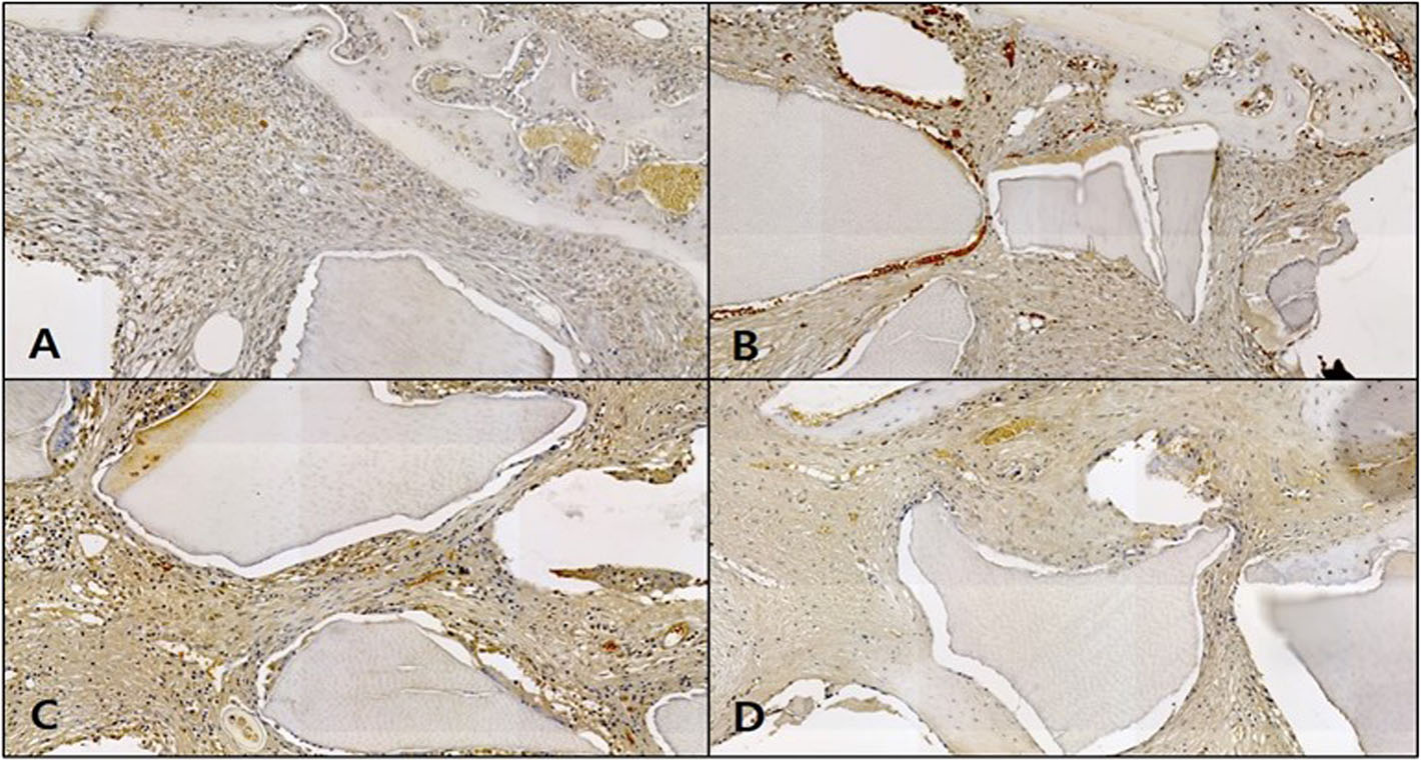
Microphotograph at 1 week (**A**), 2 weeks (**B**), 4 weeks (**C**), and 6 weeks (**D**) after bone graft with organic matter removed particulate autogenous tooth and type 1 collagen in experimental rabbit. Osteocalcin. ×50

In the OM-removed-collagen-treated experimental group B, OSC expression was absent in weeks 1 and 2, then it appeared in week 4, while in week 6, OSC expression in neonatal bone tissue was observed although the level of expression was weak (Fig. 15).

## Discussion

In the reconstruction of hard tissue defects, the most ideal bone graft material is the autogenous bone. The autogenous bone exhibits osteogenic, osteoinductive, and osteoconductive potentials simultaneously, while it causes no immune rejection and has the benefit of rapid healing. However, the downside is the limitation of collectible amount and the need for a donor site [17]. To resolve the limitations of allogenic, heterologous, and alloplastic bones, there is an ongoing research and development on various methods, and furthermore, it is necessary to develop an ideal bone graft material like the autogenous bone [4–6].

Recently, a bone graft material based on extracted autogenous tooth has been developed and applied to clinical practice, as the material overcoming the demerits of allogenic, heterologous, and alloplastic bones. The autogenous tooth bone graft material (AutoBT) has been developed based on the close correspondence between dental components and bone components. The elaborate collagen structure of tooth provides an ideal environment for the differentiation and proliferation of cells essential in new bone formation, such as bone mesenchymal stem cells and osteoblasts. As an autogenous tissue, AutoBT ensures excellent biocompatibility without immune rejection, and together with the fact that there is no concern for disease infection or need for a donor site [2, 3], the emotional rejection patients used to display before allogenic or heterologous bone graft can be eliminated. AutoBT has also been reported to exhibit not only osteoconductive but also osteoinductive potential [9–15], and it can be produced in diverse sizes and forms to allow easy application.

The early research on the use of tooth as a graft material mainly focused on inorganic matter, especially on hydroxyapatite. The high-temperature treatment of tooth led to the collection of hydroxyapatite and creation of organic matter-removed particulate dentin that was reported to be suitable as a graft material for increasing the alveolar bone. The burnout of tooth removes the organic matter to leave the inorganic hydroxyapatite and β-TCP as the main components, and this is referred to as particulate dentin. The graft of particulate dentin mediates rapid resorption and substitution by a bone tissue at an early stage, followed by a pattern of reduced resorption. This is due to the composition of particulate dentin; the early rapid resorption is due to β-TCP and the later slow resorption pattern is mainly due to hydroxyapatite. Such burnout method resembles the method of processing heterologous bone, and a high-temperature treatment of tooth forms high crystalline carbonic apatite to cause low resorption in the body, which may have an adverse effect on bone formation [10, 11].

Hydroxyapatite is the main component of the inorganic matter that composes 95% of enamel and 70-75% of dentin, and it plays a crucial role in maintaining the volume of bone graft material and in osteoconduction. The organic matter in dentin is approximately 90% collagen, mostly type I collagen, which plays a key role in calcification. The rest of the organic matter comprises non-collagenous proteins, carbohydrate, lipid, citrate, and lactate [9]. It is known that proteins have various bone growth factors including BMPs that play significant roles in the osteoinductive potential of AutoBT [14].

Recent studies have focused on the method of demineralization that removes the inorganic matter among the dental components of AutoBT while leaving the organic matter. Rather than the heat treatment method to produce particulate dentin, a reagent is used to demineralize the tooth to remove the inorganic matter, with the product being used in a form of powder or block. The reduced proportion of inorganic content in the graft material induces neonatal bone formation as well as the process of resorption and substitution of the grafted material by a bone tissue, which may improve the beneficial effect of the graft material on bone regeneration. However, the rapid resorption of demineralized graft material may lead to inadequate ability to maintain the space and a drawback of excessive resorption of the graft material [18].

This study thus set out to examine the osteogenic potential of AutoBT after the removal of organic matter in the dental tissue using sodium chlorate (NaOCl), ethyl alcohol, and chloroform, as well as that after the supplementary addition of type I collagen constituting over 90% of the organic matter of tooth. The in vitro cytological experiments showed that the affinity for cells was the highest in the control, followed by the OM-removed-collagen-treated group and the OM-removed group, while cell adhesion morphology also showed a decrease in adhesion in the above order. A similar result was obtained for the cell proliferation rate. This is a consequence of cell induction effect from the addition of type I collagen, and combining AutoBT with the addition of type I collagen was shown to have led to enhanced osteoinductive potential. The estimated degree of mineralization showed a contrasting result, where comparatively higher values were obtained for the OM-removed group and the OM-removed-collagen-treated group than the control group. This is determined to be due to the increased bone density from the removal of organic matter, which is likely to help AutoBT to enhance the osteoconductive potential and maintain the bone volume.

In addition, this study monitored the changes in rabbit calvarial tissue after having created four same size defects (8 mm) and applied AutoBT without OM and type I collagen. In the control, no treatment was given to the defect site, whereas in the experimental group A, an independent treatment of a graft using AutoBT without OM was given to the defect site, and in the experimental group B, a combined treatment of a graft using AutoBT without OM and a simultaneous addition of 100 mg/ml type I collagen was given to the defect site. At the end of weeks 1, 2, 4, and 6, the rabbits were sacrificed and the levels of bone formation were comparatively observed. Histopathologic and immunohistochemical analyses were also conducted.

The result of histopathologic analysis showed that, in the experimental group grafted with AutoBT without OM, the formation of connective tissue and blood vessel was observed around the graft material at an earlier period than the control, while neonatal bone formation was also detected at an early stage. In the experimental group grafted with AutoBT without OM and additionally administered with type I collagen, more intense vascularization was observed at an earlier period, compared to the other experimental group and the control, with neonatal bone formation detected at an earlier period. In week 4, a characteristic collagen expression in the compact connective tissue immersing the graft material began to be detected, and the expression intensified toward week 6. Also, in week 6, a high level of neonatal bone formation could be seen, compared to the other experimental group and the control, with a large number of osteoblasts observed in the area around the graft material. These findings indicated that, compared to the control, the experimental group grafted with AutoBT exhibits neonatal bone formation at an earlier period, and the additional collagen further promotes the formation. In week 6, the experimental group that received the treatment combining AutoBT and type I collagen led to the detection of a larger number of osteoblasts, which may implicate the possibility of further bone formation at a later stage. To conclude, the combined treatment of AutoBT without OM and type I collagen facilitates the neonatal bone formation while increasing the level of bone formation further, whereby the advantages of AutoBT as a graft material are maximized.

This study went on to conduct immunohistochemical analysis to monitor the expressed level of osteocalcin (OSC), a protein among BMPs, in the tissue of the control and each experimental group, in order to examine the effects of AutoBT without OM and type I collagen treatment on bone formation. OSC is a non-collagenous protein engaged in the accumulation of inorganic matter produced in bone or tooth [19]. Previous studies have found OSC expression in osteoblasts of the periodontal ligament close to the alveolar bone upon tooth movement in rats, which was characterized by the expression of OSC in the bone formation area but a lack of OSC in the resorption area [20]. In humans, OSC is found uniquely in osteoblasts and dentinoblasts, and considering this characteristic, OSC expression can serve as a marker for determining osteogenic potential in the expressed area [21–23]. In this study, OSC expression was not observed in the control, but the expression was detected in both experimental groups A and B in week 4. The expressed level was, however, far higher in the experimental group B with additional type I collagen treatment. This was indicative of the strong osteogenic potential by week 4 upon the combined treatment of AutoBT without OM and type I collagen, and the fall in OSC expression in week 6 showed that neonatal bone formation was accompanied with a gradual decrease in osteogenic potential.

The findings of this study suggested that AutoBT without OM played a sufficient role as a bone graft material in the spatial retention and osteoconduction, and that the osteogenic potential was enhanced by additional type I collagen treatment. Nevertheless, the difference in the ability as a bone graft material was not significant, which was presumed to be due to the low level of other bone morphogens such as BMPs. Thus, it is necessary that further studies focus on whether the supplementary addition of bone morphogens and AutoBT without OM could carry out the role of a mediator.

In this study, a graft material based on human autogenous tooth rather than rabbit tooth was used. For the animal experiments, human autogenous tooth bone graft material was used unlike the previous studies where animal tooth was used, because it was predicted that the graft of pre-treated human AutoBT without OM as in conventional cases of allogenic or heterologous bone graft, would not cause immune rejection in rabbit calvaria, in addition to the issue of nutrient supply due to extraction [24]. Based on the findings of this study, AutoBT without OM and the addition of type I collagen is likely to play a sufficient role as a bone graft material in clinical practice. Nevertheless, studies should continue to focus on the immune rejection upon an allogenic graft of AutoBT without OM.

## Conclusion

In present study, we were to determine the osteogenic potential of AutoBT from which organic matter of dental tissue was removed using sodium chlorate (NaOCl), ethyl alcohol, and chloroform through the use of the autogenous tooth bone maker.

1. The affinity for cells was highest in the control group, followed by OM-removed-collagen-treated group, and OM-removed group in vitro. This is a result of the cell-inducing effect of type I collagen treatment, and it has been shown that AutoBT combined with the addition of type I collagen can enhance the bone induction potential. The estimated degree of mineralization showed contrasting results with the OM-removed group, the OM-removed-collagen-treated group showing relatively higher values than the control group.
2. In the histopathological analysis, compared to the control group, neonatal formation was observed in the experimental group in which AutoBT was transplanted without OM and the experimental group in which AutoBT was grafted without OM and type I collagen was additionally administered, compared to the control group and other experimental groups, and also osteocalcin (OSC) was found to be slightly higher in the group receiving AutoBT and type I collagen.

Taken together, AutoBT without OM can be seen a graft material that exerts a positive effect on the grafted area by enhancing the osteogenic potential and the additional administration of type I collagen further enhances the osteogenic potential. Notably, a positive effect on neonatal bone formation was shown 4 weeks after the bone graft, suggesting a contribution to the possible reduced bone resorption that may arise in the long run after the graft, thereby ultimately leading to increased bone volume.

## Data Availability

All data generated or analyzed during this study are included in this published article.

## Abbreviations

AutoBT: Autogenous tooth bone graft material
OM: Organic matter
SEM: Scanning electron microscopy
EDS: Energy dispersive X-ray spectroscopy
OSC: Osteocalcin
